# The Potential for Harvesting Energy from the Movement of Trees

**DOI:** 10.3390/s111009275

**Published:** 2011-09-28

**Authors:** Scott McGarry, Chris Knight

**Affiliations:** Commonwealth Scientific and Industrial Research Organisation (CSIRO), P.O. Box 330, Newcastle, NSW 2300, Australia; E-Mail: scott.mcgarry@csiro.au

**Keywords:** energy harvesting, energy scavenging, tree energy, tree movement, tree sway, wireless sensor network, sensor node, wind energy

## Abstract

Over the last decade, wireless devices have decreased in size and power requirements. These devices generally use batteries as a power source but can employ additional means of power, such as solar, thermal or wind energy. However, sensor networks are often deployed in conditions of minimal lighting and thermal gradient such as densely wooded environments, where even normal wind energy harvesting is limited. In these cases a possible source of energy is from the motion of the trees themselves. We investigated the amount of energy and power available from the motion of a tree in a sheltered position, during Beaufort 4 winds. We measured the work performed by the tree to lift a mass, we measured horizontal acceleration of free movement, and we determined the angular deflection of the movement of the tree trunk, to determine the energy and power available to various types of harvesting devices. We found that the amount of power available from the tree, as demonstrated by lifting a mass, compares favourably with the power required to run a wireless sensor node.

## 1. Introduction

### 1.1. Background

The use of sensor networks for remote monitoring within forests is becoming widespread. Use of these networks in forests is occurring for detection and monitoring of forest fires [1], monitoring of various environmental data [2], monitoring wildlife passages [3] and much more.

We conceived the idea of harvesting energy from tree movement due to the problem of energy supply that currently affects users of remote wireless sensors nodes located under the canopies of trees in forests [2,4]. Remote wireless sensors have predominantly been powered by batteries, recharged by photovoltaic cells (solar cells). However, without access to sunlight, due to the shading effect of the trees or forest canopy (as low as 2.4% incident light levels within some forests [5]), solar recharging of batteries becomes difficult, if not impossible.

Within densely wooded forest, access to wind power is also limited. Wind speeds under the canopies are far lower than those above the canopy; as much as 1/10 to 1/100 the velocity at 1.3 m above ground compared to above the canopy [6]. This is due to the trees themselves absorbing much of the wind energy, and dissipating it as wind vortices, movement and heat. Conceptually, if some of the movement energy of a tree could be harvested it may be possible to recharge batteries to power a remote wireless sensor node, though the use of a movement energy harvesting device. The device would encompass an energy harvesting transducer to convert movement energy into electrical energy. The magnitude of energy available from tree movement is not well known, and we seek to address this issue.

### 1.2. Related Work

It appears that little investigation into converting the mechanical energy of tree movement to electrical energy has been previously performed. No published articles on such research exist to the authors’ knowledge, although a number of patents and patent applications for machines to convert “natural forces”, including tree movement, do [7–9], in particular [10] claims up to 1 kW might be available from a 20 m tall tree.

Some work has been performed to harvest energy from trees using other methods, including the Voltree Bioenergy Harvester [11], which the company claims “*converts living plant metabolic energy to useable electricity*”. This method of energy harvesting, using voltage differences within a living tree, has been described in publication [12].

A number of other mechanisms exist [13] that may convert energy in a tree system to useable energy. These include thermoelectric devices [14–16], piezoelectric vibration harvesting [17] and wind driven micro turbines [18–20]. Each of these mechanisms offers advantages and disadvantages.

The thermoelectric based systems are currently characterised by low efficiency, particularly at the 1–5 K thermal difference offered across the internal to external structure of a tree. Piezoelectric based vibration harvesting, which is effectively a higher frequency version of the mechanisms discussed in this paper, typically need frequencies in the range of 100+ Hz to be efficient. Wind driven micro turbines, of the sort noted in the references, have start up speeds of over 3 m/s, which are rare under dense tree coverage.

### 1.3. Presented Work

We offer a number of possible methods by which energy may be extracted from tree movement, and the investigation into the amount of energy available for energy harvesting from tree movement, to power a wireless sensor node. These methods include energy harvesting via the horizontal acceleration of the tree, the lean angle of the tree and the force/displacement of the tree, as demonstrated by having the tree lift a mass. Device design methodologies to extract energy from these different aspects of tree movement are explained. We found that energy harvesting using a device that could exploit the force/displacement of the tree showed a greater potential than the other proposed methods. We also present a simple model of a tree with the goal to estimate power dissipation by any tree, and energy available at a given point on the tree trunk.

## 2. Proposed Movement Energy Harvesting Techniques

To capture movement energy from trees, a number of possible methods of operation for a device have been conceived. These methods can be broadly categorised into ‘inertial mass’ and ‘tethered’ approaches. Either of these approaches may ultimately employ the use of electromagnetic or Piezo transducers.

### 2.1. Movement Energy Harvesting Employing an Inertial Mass

**Figure 1.**
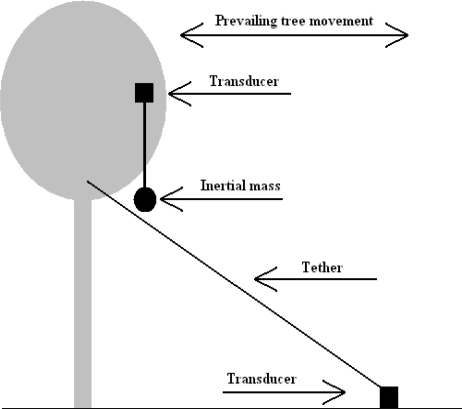
‘Tethered’ and ‘Inertial Mass’ general arrangements.

The inertial mass method of movement energy harvesting requires a mass to be suspended and connected to a transducer, which then attaches to the tree, as shown in Figure 1.

As the tree sways, the mass may move relatively to the tree due to either relative acceleration, change in orientation with respect to gravity, or possibly resonant oscillation. The transducer will operate as a result of the relative movement between the inertial mass and the tree. The shaker style flashlight and the Seiko Kinetic™ range of watches are both examples of inertial mass movement energy harvesting.

Inertial mass movement harvesters may be arranged in a number of ways, such that the inertial mass has at least one degree of freedom of movement with respect to the tree. It may also require some form of restoring force to return the mass to the starting position after movement.

#### Principles of Operation (Inertial Mass)

The methods by which this may be accomplished include a pendulum that swings about a horizontal axis, which is restored to vertical (or near vertical) by gravity after side to side movement has ceased; a pendulum arranged to swing about a vertical axis, possibly with a spring to return to a known starting position; a mass allowed to travel back and forth linearly along an axis (say in a tube) arranged horizontally or perhaps another orientation.

To drive a linear inertial mass harvester requires some amount of acceleration. That is, to provide a *force* on the transducer the *tree must accelerate relative to the inertial mass*, thus providing relative movement. It is the combination of force and displacement (movement) that will provide energy to the harvester. From Newton’s Second Law, net force, or the force acting on the transducer, is a product of mass and acceleration:

(1) $$F_{\mathrm{NET}}=m\cdot a$$

Thus, the net force applied to the transducer, if linear in action, is a product of the magnitude of the inertial mass (*m*), and the magnitude of the acceleration (*a*) of the point of the tree to which the transducer is attached. The initial ‘linear’ movement of a pendulum based system can also be approximated by the equation whenever the movement of pendulum mass remains close to parallel to the movement of the tree (*i.e.*, pendulum arm is close to perpendicular to movement).

Further, an inertial mass energy harvester may be driven not by the acceleration of the tree but by *force due to gravity*. That is, if a tree leans over slowly, such that any acceleration is negligible, the inertial mass may still move relatively to the tree due to the change in angle of orientation of the harvesting device relative to the earth. For example, in a horizontal axis pendulum based system, if the tree leans in the same plane in which the pendulum swings, the force of gravity will act to force the pendulum to vertical, because the transducer is rotating relative to earth due to the lean angle of the tree. In the case of a horizontally arranged linear device, if the axis of operation is tipped away from horizontal, the force of gravity will act to slide the mass down the inclined axis. The term for acceleration in the above equation, (*a*) is exchanged for a component of acceleration due to gravity (*g*), based on the angle through which the tree has leaned (*θ*), such that, for a linear system or rotational device:

(2) $$F_{\mathrm{NET}}=m\cdot g\cdot \text{sin}\left(\theta \right)$$

Having provided a force to the transducer, the combination of the magnitude of the displacement or distance moved (Δ*s*), of the inertial mass relative to the tree, and the actuating force (*F_NET_*) will determine the energy input (*E_MOV_*) to the inertial mass system. That is, energy is the integral of force as a function of position, with respect to the change in position (displacement):

(3) $$E_{\mathrm{MOV}}=\int F_{\mathrm{NET}}\left[s\right]\mathrm{ds}$$

For a constant acceleration, and therefore constant force, the above equation can be simplified for any period of time to:

(4) $$E_{\mathrm{MOV}}=F_{\mathrm{NET}}\;\cdot \;\Delta s$$

For a pendulum based harvester, and assuming small horizontal tree displacement, the energy input into the system can be determined as a function of the rise/fall (*h*) of the pendulum mass relative to the attachment point on the tree:

(5) $$E_{\mathrm{MOV}}=m\cdot g\cdot h$$

That is, if a pendulum device is actuated purely via the lean angle, *θ*, of the tree the energy for a pendulum rotating around a horizontal axis, where *L_PEND_* is the effective length of the pendulum arm is:

(6) $$E_{\mathrm{MOV}}=\left({\mathrm{mgL}}_{\mathrm{PEND}}\right)-\left({\mathrm{mgL}}_{\mathrm{PEND}}\;\cdot \;\text{cos}\left(\theta \right)\right)$$

It can be seen from the above equations that the greater the inertial mass (*m*) the greater the movement energy available (*E_MOV_*) to the harvester. However, the tree itself will be limited in how much mass it can support at any given point. Further, for a pendulum based system, there will be a limit to the practical effective length of the pendulum.

### 2.2. Movement Energy Harvesting via a Tethered Transducer

The ‘tethered’ method of operation requires a transducer to be attached at two points. These points can be one of a number of combinations e.g., a tree and the ground, two neighbouring trees, or two points within the same tree (between branches or from a branch to the trunk). See Figure 1 showing a tether between the tree and the ground.

#### Principles of Operation (Tethered)

A tethered transducer is acted upon directly by two opposing forces; between a point on a tree and some other. Therefore the reaction force (*F_TRAN_*) at the transducer is the resistance of the transducer to the force acting upon it. Additionally, the distance through which the transducer acts, or is displaced (Δ*s*) will also be as a direct result of the distance the tree (or trees) moves from some arbitrary starting location. That is, the energy into a tethered transducer based harvester takes the same form as Equation (3), except that force (*F_TRAN_*) is simply the resistance force in the tether, rather than a function of mass or acceleration such that:

(7) $$E_{\mathrm{MOV}}=\int F_{\mathrm{TRAN}}\left[s\right]\mathrm{ds}$$

It should be noted that if the resistance force offered by the transducer is greater, the distance the tree can displace will be minimised due to the force acting against it. That is for a small reaction force, a greater displacement can occur because little is acting to stop the tree from moving, and for a large reaction force little displacement can occur. Again, to maximise energy input into the harvester, at a given point on a tree, there will be an optimum level of resistance force compared to movement, as the tree sways.

## 3. Methods to Determine the Magnitude of Energy in a Tree

Methods to determine how much energy at a given point on a tree trunk were investigated and are described. A model of a tree is presented to provide a basis for further calculations.

### 3.1. An Energy Model of a Tree

**Figure 2.**
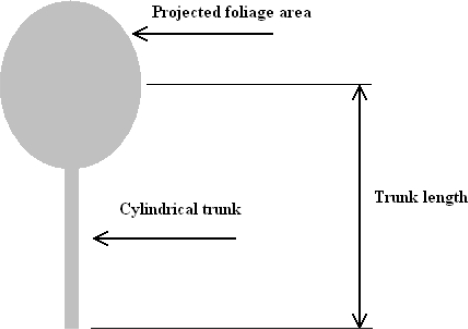
‘Lollipop’ model of a tree.

As a starting point to determining how much power and energy might be dissipated by a tree, a simple aerodynamic model of a tree has been devised and analysed. The modelling was based on conventional aerodynamic and mechanical/material science theory. See Figure 2 showing the ‘lollipop’ model of a tree.

The intention was that the model, if verified by experimentation, would allow predictions to be made about energy dissipation by any tree at its trunk (or branch if developed further) given known wind conditions and various dimensions of the tree.

The study of fluid dynamics has shown that the force acting on an object due to laminar fluid flow can be given by:

(8) $$F_{D}=\frac{1}{2}\left(\rho \cdot v^{2}\;\cdot \;\mathrm{Cd}\;\cdot \;A\right)$$

where:

*F_D_* is the force of drag, which is by definition the force component in the direction of the flow velocity,
*ρ* is the mass density of the fluid,
*ν* is the velocity of the object relative to fluid (or velocity of fluid relative to object),
*A* is the reference area, (the projected area of foliage as ‘seen’ by air flow) and
*Cd* is the drag coefficient—a dimensionless constant.

From the above equation, the force acting on the tree can be determined for any given wind speed, if the projected area of the foliage (*A*) and the drag coefficient (*Cd*) are known. This assumes that the projected area of the foliage (*A*) is constant, when in fact it changes with wind speed. The equation therefore supplies a conservative upper limit on the magnitude of the force due to the wind. Additionally, determining the value of *Cd* of any given tree is difficult; in any case the concept of the model was conceived as a starting point or rough estimate of energy absorption by a tree.

From the force acting on a tree, the distance through which the tree sways can be determined from the mechanical properties of the tree trunk. That is, if the properties of the tree trunk are known, and the force applied is known, the elastic deflection of the tree trunk can be determined. Once the deflection is known Equation (7) can be used to determine the energy absorbed by the deflection of the trunk due to a given wind speed (or gust).

Further, if the force acting on the tree is known, the magnitude of wind power can be determined from the following:

(9) $$P_{W}=F_{D}\;\cdot \;v$$

where *P_W_* is the wind power (rate of wind energy) required to maintain a given force (*F_D_*) on the tree.

To find the deflection of the model tree trunk, the ‘spring rate’ (*k*) of the model tree trunk must be known. Beam deflection theory (Euler Bernoulli) can then be used to calculate deflection. The small deflection (*y*) in a constant section cantilever beam, with a point force (*P*) applied at the free end of the beam can be given by:

(10) $$y=\frac{\left({\mathrm{PL}}^{3}\right)}{\left(3\mathrm{EI}\right)}$$

By definition, the spring rate (*k*) is:

(11) $$k=\frac{P}{y}=\frac{\left(3\mathrm{EI}\right)}{L^{3}}$$

where:

*k* = spring rate of beam (tree trunk),
*y* = linear deflection of the beam; deflection in the horizontal plane for tree model,
*P* = force applied = *F_D_* in the case of the tree model,
*L* = length of trunk,
*E* = Young’s Modulus (or Elastic Modulus),
*I* = second moment of area of the cross section of the beam.

Further, the value *I* can be found for a solid circular section, from:

(12) $$I=\frac{{\pi D}^{4}}{64}$$

where *D* = is the ‘mean effective’ diameter of the trunk, to approximate a cylindrical section in bending. The mean effective trunk diameter is the diameter of a constant section beam which deflects similarly to the tree trunk (which is tapered), for the same applied force.

The value for Young’s Modulus (*E*) for a living tree or ‘green’ timber, has been found to be highly correlated to the density of the timber. Dinwoodie [21] gives a plot for over 200 species tested, showing the correlation between Specific Gravity (density of wood relative to density of water) and Young’s Modulus to be a line of best fit with the following form:

(13) $$E_{G}=13.517\;\cdot \;G^{0.9796}$$

where:

*G* = specific gravity (density of wood relative to density of water),
*E_G_* = E (10^−9^) = Young’s Modulus (measured in GPa rather than standard units of Pa).

To verify the model, a local, easily accessible tree was chosen for experimentation.

### 3.2. The Experimental Tree

**Figure 3.**
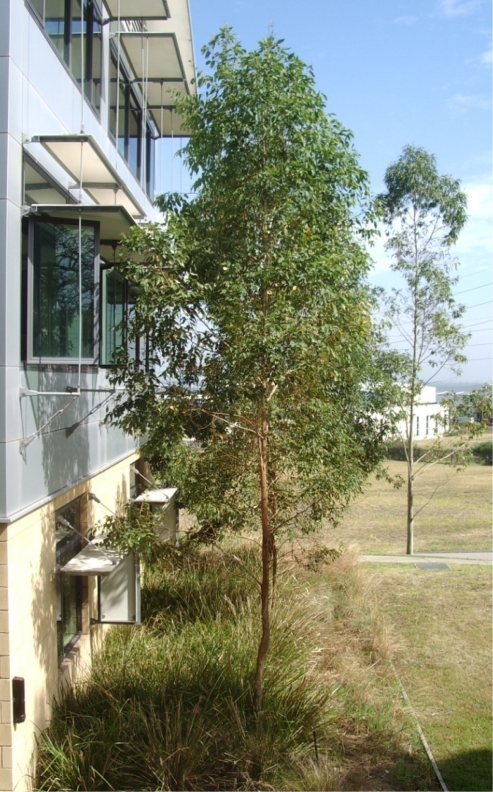
Photograph of the experimental tree.

A eucalypt tree (Figure 3) was chosen for experimentation. Trunk dimensions were measured and recorded. On a calm day, applied force and resultant displacement measurements were conducted at a point on the tree trunk approximately at the centre of the foliage (from side view), to find the effective spring rate of the trunk (*k*), at that point. The force measurement was performed using a mass calibrated spring balance attached to the tree trunk via a stiff clamp and inelastic nylon cord. The displacement of the cord, at the point the force was applied, was measured using a horizontally arranged tape measure.

The tree used for experimentation was chosen due to its proximity to an office building and opening windows. This position was not considered ideal, but offered a number of critical advantages. These include:

- Safe access to the tree for attachment of instruments and measuring equipment, including sensitive electronics, which would have been difficult to otherwise achieve.
- Ability to mount a video recorder and a computer next to the experiment.
- The equipment was out of the weather in a secure location with power.

This position was not considered ideal for determining accurate wind speed measurements due to the wind shading and wind vortices resulting from the nearby building. Importantly, the experimental tree was considered representative of trees expected to be candidates for this style of energy harvesting; including that likely trees chosen would be subject to turbulent conditions.

To be able to verify all results from the model, a number of data were required. These included the difficult to obtain values for *Cd* (drag coefficient for the tree), *A* (reference or projected area of foliage) and *D* (mean effective diameter of tree trunk). They also included those that could be measured or obtained from literature including maximum trunk diameter (*D_MAX_*), minimum trunk diameter (at approximate centre of foliage, *D_MIN_*), trunk length (*L*) and Specific Gravity (or density) of the timber (*G*).

The projected area for this tree (*A*) was determined by photographic analysis. This process was performed by printing an image of the tree onto a finely spaced grid, and then counting the number of ‘pixels’ which were determined to have more foliage than background present. The number of pixels counted was multiplied by the area represented by each pixel (99.2 mm × 86.1 mm). The projected area of the tree is considerably less during a wind, due to leaves and small branches leaning in the direction of airflow. Thus the projected area should be seen as an upper bound value, rather than an accurate indicator. Additionally, the projected area may change depending on the angle from which the tree is viewed, and therefore is dependent on the direction from which the wind blows.

The value for the drag coefficient of the tree is unknown. Little information exists with reference to drag coefficients of trees, however Munson *et al.* [22] suggest that for wind speeds up 10 m/s, the coefficient of drag of a tree is about 0.43. For calculations, this value was used.

To obtain the specific gravity (*G*) of the timber of the tree trunk without destructively testing the timber, the species was required. It was known that the tree was a native eucalypt hardwood, for which the range of obtainable values is from 1,000 kg/m^3^ to 1,200 kg/m^3^. As such, it was decided to use 1,100 kg/m^3^ for calculations, or a specific gravity (*G*) of 1.1. From this value for specific gravity Young’s Modulus (*E*) was calculated using Equation (13).

To be able to predict the lean angle of any tree due to a given applied force using simple constant section beam analysis, the mean effective trunk diameter (*D*) is required. It was expected that the mean effective diameter would be close to the small diameter (*D_MIN_*) of the trunk, as the majority of bending/deflection occurs at the small end of the tapered trunk. This was corroborated by experiment. To determine the mean effective diameter of the tree trunk a number of steps were taken. The force and displacement measurements were used to find the spring rate (*k*) of the experimental tree trunk, in bending. The point at which force and displacement readings were obtained is where the trunk length (L) was measured to, from the base of the tree. The force recorded, using a spring balance, was 61 N, and the displacement was 210 mm. The value for *k* was found through use of Equation (11). Rearrangement of Equations (11) and (12) gave the following equation:

(14) $$D^{4}=\frac{\left(64\cdot k\cdot L^{3}\right)}{\left(3\cdot E\cdot \pi \right)}$$

From the above, *D* was determined to be 0.056 m (56 mm). A summary of all values of interest for the experimental tree are listed in Table 1.

### 3.3. Method to Determine Energy Available for a Tethered Harvester Using the Experimental Tree

**Figure 4.**
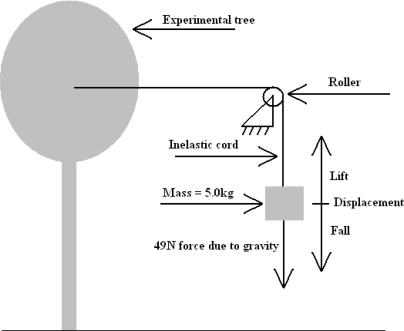
Tethered harvester experimental arrangement.

A test was conducted which involved attaching, and hanging, a known mass to an inelastic cord, which was then passed over a roller to attach horizontally to a clamp rigidly attached to the tree trunk. See Figure 4 showing the arrangement of components.

**Figure 5.**
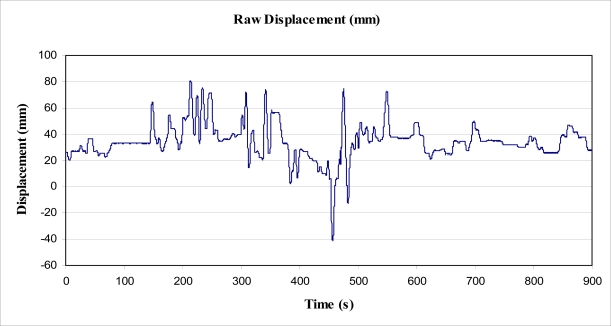
Displacement of suspended mass, *versus* time.

The cord was marked with tape above the mass (of 5.0 kg), and a graduated placard placed behind the weight and marked cord. A video recorder was then set up to record the position of the marker in relation to the graduated placard, to record the positional change over a period of 15 min (900 s). The video recorder sampled at a rate of 10 Hz (10 frames per second). The mass was not allowed to touch the floor during the experiment, and was, therefore, always applying a 49 N force to the tree trunk. The video data was analysed with video processing software to plot the motion of the mass on the cord against time. From this, the magnitude of the energy required to raise the mass for each sway of the tree was determined, and the subsequent power output over time calculated. The data obtained from this experiment is shown in Figure 5.

At the time of the experiment, the wind conditions were observably windier than average for this site (Beaufort 2). This corresponded to an estimated wind of 4 on the Beaufort scale of wind force (5.5 m/s to 7.9 m/s).Unfortunately, no correlating wind data for the time of the experiment exist. It was noted at the time of the experiment that the roller was not frictionless, and that a small amount of energy was required to overcome the friction of the roller on its bearing shaft. This was not considered to be of concern in determining ‘ballpark’ values for preliminary calculations.

This experiment was able to measure energy from movement in one axis, *i.e.*, the component of tree movement parallel to the cord. No other component of movement energy was captured by this experiment.

### 3.4. Method to Determine Energy Available for an Inertial Mass Harvester Using the Experimental Tree

As explained in Section 3.1.1, an inertial mass harvester requires acceleration or effective acceleration (due to gravity) to operate. To determine the potential for such a device to operate in a tree, a further experiment was conducted, and further analysis of existing data performed.

#### 3.4.1. Actuation via Horizontal Acceleration of a Point on the Tree

**Figure 6.**
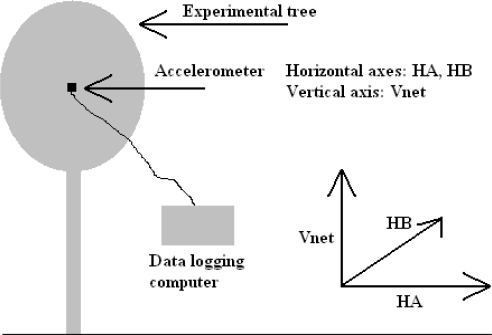
Inertial harvester experimental arrangement.

The experiment involved placing a 3 axis accelerometer in the tree, attached to the same clamp in the same position as the first experiment (as shown in Figure 6), to record acceleration rates directly. This device was less than 100 g in mass and was considered to have a negligible effect on the tree, allowing the maximum acceleration rates to be realised by the tree trunk. The accelerometer was arranged such that one of the 3 axes (Y axis) was as close horizontal as possible.

Data was collected from the accelerometer over a period of 9 h at 2 Hz sampling frequency, of which the first 273 s were sampled at 32.636 Hz (or 0.0306 s intervals). The experiment was started on an afternoon that was observably not as windy as the day of the displacement experiment, but still determined to be represented by a 4 on the Beaufort scale of wind force.

**Figure 7.**
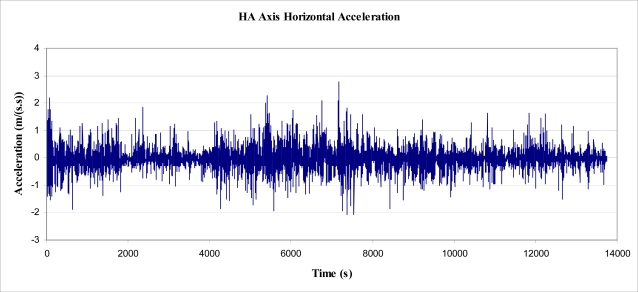
HA Axis Acceleration, as recorded by an accelerometer.

Using basic trigonometry, the raw data were transformed into 3 orthogonal axes, labelled HA, HB and Vnet. Axes HA and HB were both arranged horizontally relative to gravity, and Vnet arranged vertically. It should also be noted that Vnet was the difference between the magnitude of acceleration in the vertical direction and acceleration due to gravity, *i.e.*, the net vertical acceleration. The peak magnitude of Vnet was 0.5 m/s^2^, and, as a result, was not considered worthy of evaluation. The following figure, Figure 7, shows a plot of acceleration in the two horizontal axes, for the first 13,753 s of recorded data.

Figure 7 was created based on the assumption that the orientation of the accelerometer did not change with respect to gravity. What was not known was the magnitude of the component of acceleration in HA or HB, due to the leaning of the tree. Further analysis was performed to investigate the possible contribution of acceleration due to gravity, caused by tree lean.

It should be noted that a second analysis using the displacement data, as obtained by methods described in Section 3.3, was undertaken. This data was differentiated to find acceleration and thus energy. In the interests of brevity this analysis is not included as it provided a lower bound for the predicted power and energy available to be harvested and is of academic interest only for a prediction of maximum output.

#### 3.4.2. Actuation via Lean Angle of the Tree

An inertial mass based movement energy harvesting device has the potential to operate due to the lean angle of the tree, and therefore due to a force due to gravity. That is, rather than relying on the acceleration of the tree in relation to an inertial mass to impart a force (or torque), the force can be applied directly by gravity due to the reorientation of the attachment point (tree) with respect to the earth.

To determine the magnitude of the lean angle of the experimental tree, the data gathered as outlined above, and displayed in Figure 5, was further analysed. That is, the angle of lean of the tree was determined, for any given horizontal displacement, based on the already determined properties of the tree.

It has been previously shown that the tree trunk could be modelled as a vertically arranged cylindrical beam, and an equivalent ‘mean effective diameter’ found. Using some of this information (namely beam length) the angle of the beam, at the point where the displacement measurements were obtained, was calculated. Beam theory was again used to perform the calculations. It can be shown that the slope (*y’*) of a cantilever beam experiencing a point load at the free end can be given by:

(15) $$y\prime =\frac{\left({\mathrm{PL}}^{2}\right)}{\left(2\mathrm{EI}\right)}$$

Substitution of Equation (10) into Equation (15) gives the much simpler equation of:

(16) $$y\prime =\frac{3y}{2L}$$

To determine the angle of lean (*θ*) due to the slope (*y’*), simple trigonometry gives:

(17) $$\theta =\text{arctan}\;\;\;\;y\prime =\text{arctan}\;\left(\frac{3y}{2L}\right)$$

**Figure 8.**
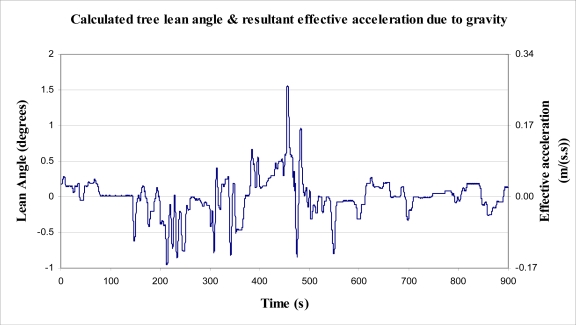
Calculated Lean Angle and resultant effective acceleration.

The displacement data, shown in Figure 5, was analysed to determine the lean angle of the tree (in degrees, for readability). The results are shown in Figure 8, below, by reading the left side axis.

From a given lean angle, the component of the force due to gravity can be determined, again using simple trigonometry. Further, this component of gravity can be described as an acceleration, rather than as a force, to allow comparison to the horizontal acceleration plot (from Section 3.4.1). That is, the component of acceleration due to gravity (*a_g_*), required to cause a pendulum angular displacement of *θ*, can be given by:

(18) $$a_{g}=g\;\cdot \;\text{sin}\left(\theta \right)$$

Again, refer to right side axis in Figure 8 showing the effective acceleration due to gravity as a result of tree lean.

## 4. Power and Energy Analysis and Results

### 4.1. Wind Power and Energy Dissipation by a Tree

**Figure 9.**
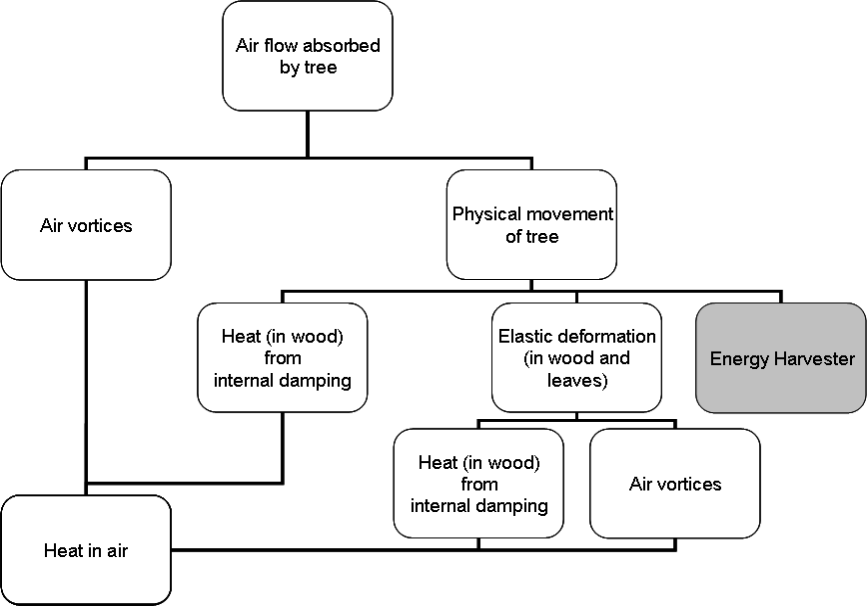
Wind power/energy dissipation breakdown.

The breakdown of the energy or power imparted by wind upon a tree is shown in Figure 9. Also shown in Figure 9 is where an energy harvesting device sits in relation to the energy dissipation. In short, all energy from the wind absorbed by the tree is ultimately dissipated as heat in air via a number of pathways. The aim of the movement energy harvester is to extract energy from movement before it is converted to further elastic energy and heat in the wood. To help predict the amount of energy available for harvesting, a breakdown of the total power for any given point on the chart is ultimately sought.

To that end, as a first step, total wind power for an average wind speed past the experimental tree has been determined. Further, elastic energy and maximum elastic power input have been estimated.

#### 4.1.1. Total Wind Power Dissipation (Modelled)

To determine the wind power dissipated by a tree for a given wind speed, the value for density of air (*ρ =* 1.2 kg/m^3^) and values stated in Table 1 were substituted, into Equations (8) and (9). For the case of a Beaufort 4 wind speed of 6.7 m/s, the power dissipated by the tree was calculated to be 496 Watts.

This value is the total power dissipated by the tree. This value includes not only the movement of the tree which would be lost as heat within the tree structure, but also any vortices created in the passing air. The proportion of the power dissipated as heat from the tree *versus* vortices within the air was unknown. Of the proportion transferred to movement energy, just a small part of that will be available to a movement energy harvesting device for energy extraction. This is due to the fact that it is envisaged that such a device will only attach to one point or area of the tree, and not be able to take advantage of the movement of the many small leaves, twigs and branches widely distributed throughout the tree’s structure.

#### 4.1.2. Elastic Energy in Tree Trunk Due to a Wind Gust (Modelled)

To determine the amount of elastic energy in the tree trunk due to one gust of wind of a given speed, Equation (8) was used to find the wind force, *F_D_*, using values as stated in Table 1 and Equation (10) was used to find the deflection of the trunk, *y* due to the wind force. From this the energy stored could be predicted by the use of Equation (7).

Further, for a linear spring of rate *k,* displaced by distance *Δs:*

(19) F=k⋅Δs

therefore:

(20) $$\begin{array}{l} E_{\mathrm{MOV}}=\int k\cdot s\;\mathrm{ds}∴\\ E_{\mathrm{MOV}}=\frac{1}{2}k{\left(\Delta s\right)}^{2} \end{array}$$

and substituting Equation (20) gives:

(21) $$E_{\mathrm{MOV}}=\frac{1}{2}F\left(\Delta s\right)$$

where:

*F* is substituted by *F_D_* for the case of a wind force acting on the tree,
*Δs* is substituted by *y* for the deflection of the tree at the point under consideration.

Substituting values into Equation (8) gives a wind force of 74 N (using the abovementioned velocity of 6.7 m/s). Substituting values into Equation (10) gives a deflection of 249 mm or 0.249 m. Substituting those values into Equation (21) provides an elastic energy content of 9.2 Joules. This value gives a reasonable representation as to the energy available for harvesting, from a given point on the tree trunk, for one ‘sway’ of the tree during a Beaufort 4 wind.

### 4.2. Power and Energy from Tree Force/Displacement (via Tether)

The data shown graphically in Figure 5 was analysed to determine the total height difference between each peak and trough within the data, for all peaks and troughs. The data was also analysed to determine the total height difference for those differences greater than 0.7 mm in displacement, to filter out ‘noise’ in the data. Each rise of the mass was added to find a total rise over the 900 s period. This rise was used to find a total energy output using Equation (4), where *F_NET_* was simply *mg*. An important assumption made for these calculations to be accurate, was that the tree was well damped, such that each period of “lift” performed on the mass was not simply the tree oscillating due to the energy from the last “fall” of the mass. Observation of the movement gave confidence that this assumption was correct.

**Figure 10.**
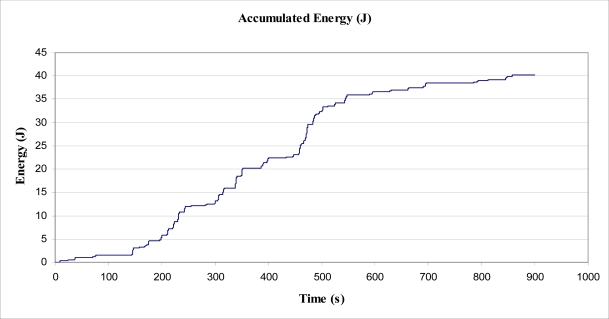
Accumulated Energy, E_MOV_ from lifting suspended mass.

Within the 15 minute period, the total value of energy required to lift the mass, by the tree, was determined to be 52 Joules, from the raw data. For individual rises of greater than 0.7 mm, the total value of energy required to lift the mass, by the tree, was determined to be 40.2 Joules, as shown in Figure 10. From this value, the average power output by the tree, over 900 s, to lift and lower the mass was calculated as 44.7 mW. This value is approximately 10,000 times smaller than the magnitude of the energy estimated to be dissipated by the tree during a Beaufort 4 wind (as estimated in Section 5.1). The value of 44.7 mW may have been greater in magnitude had a larger or smaller mass been suspended from the trunk. Additionally, the method used to determine energy obtained from the tree worked in only one (arbitrarily selected) direction, in one axis. That is, if another inelastic cord had been arranged in another direction with a mass attached, additionally to that used in the experiment, it is expected that further energy would have been obtained from the tree through work to lift a second mass.

### 4.3. Power and Energy from Tree Lean (via Inertial Mass)

Using data gathered by the experiment explained in Section 3.4.1, the lean angle of the tree was determined at each peak and trough. From each change in angle between the peaks and troughs, the energy required swinging a pendulum of mass *m*, and length *L_PEND_*, through an angle *θ* from vertical, could be determined. To determine the change in energy level for every change in lean angle (*Δθ)*, as shown in Figure 8, Equation (6) was used with *θ* substituted by *Δθ*.

**Figure 11.**
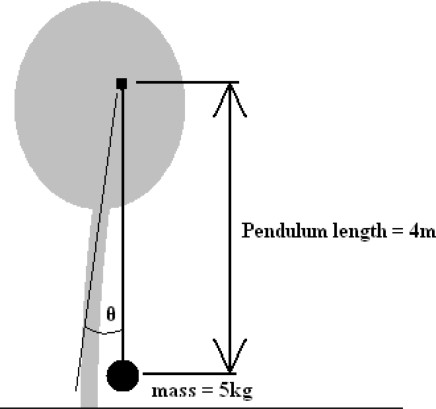
Theoretical arrangement of inertial harvester used for tree lean angle analysis.

**Figure 12.**
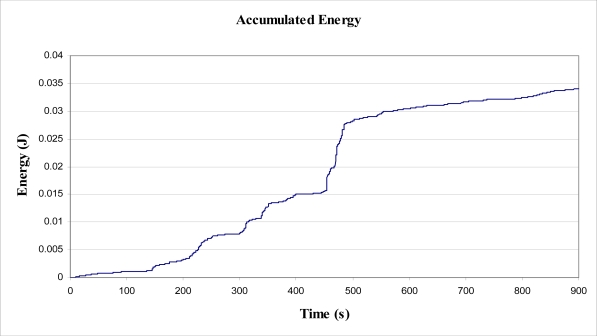
Accumulated Energy, E_MOV_, from tree lean, for inertial mass 5 kg on 4 m long pendulum arm.

As the data used were gathered via a 5 kg mass attached to the tree, it was considered prudent to use 5 kg as the value for *m* in the above calculations. From known values for *m*, *g* and *θ*, *E_MOV_* becomes a function of the pendulum length, *L_PEND_*. To maximise the energy output a value of 4 m was given to *L_PEND_* for the purpose of the calculations. The theoretical arrangement of this system is shown in Figure 11. The results of these calculations are plotted in Figure 12, below.

The running total of energy, as plotted above, results in 0.034 J after 900 s of tree sway. This equates to an average power output of about 38 μW. It should be emphasised that this figure was calculated using a pendulum length (*L_PEND_*) of 4 m, which is the maximum length as the connection point on the tree was at a height of 4 m. The mass (*m*) of 5 kg was somewhat realistic in that it was supportable by the tree.

### 4.4. Power and Energy from Tree Acceleration (via Inertial Mass)

**Figure 13.**
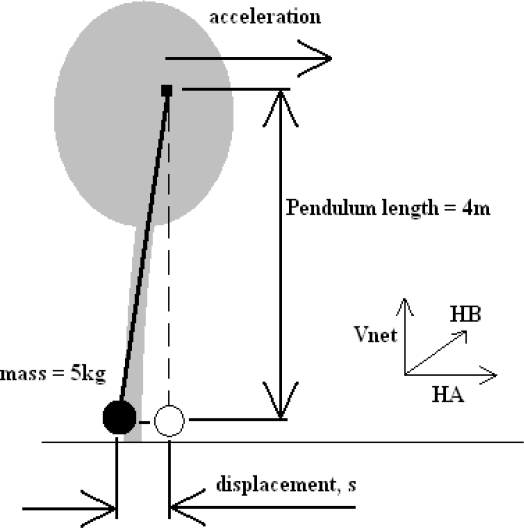
Theoretical arrangement of inertial harvester used for tree acceleration analysis.

Determination of power and energy from those data recorded by the accelerometer ultimately proved impossible to verify as correct. A process was followed with the aim of finding an upper bound figure of energy. That is, the initial aim of the experiment was to integrate the accelerometer data to find velocity, and integrate velocity to find displacement. Upon undertaking this process, a significant amount of ‘drift’ resulted. From the accelerometer data, step integration resulted in significant (unrealistic) velocities, which contributed to extreme displacements (not possible for an object fixed to ground). To counter the drift, a number of techniques were considered and implemented. Of those techniques, the process deemed to be most successful was as follows:

1. take an average, over the period of time used for analysis, of the acceleration data,
2. use the average to offset the data such that it cycles around an average of 0 m/s^2^,
3. integrate the acceleration data to find velocity,
4. produce a Fast Fourier Transformation (FFT) of velocity plot to find significance of low frequencies,
5. eliminate drift in the velocity data by filtering via a ‘high pass’ filter developed by Murphy and Robertson [23],
6. integrate filtered velocity data to find step change displacement and absolute displacement, and
7. use the step change displacement (Δs) to determine work and energy via use of
  (22) $$E_{\text{MOV}}=\text{Work}=\Sigma \left(F_{\text{NET}}\;\cdot \;\Delta s\right)$$
  substitution of Equation (1) gives:
  (23) $$E_{\text{MOV}}=\left(m\cdot a\cdot \Sigma \Delta s\right)$$

where:

*E_MOV_* = the energy obtained from the tree (or work done)
*m* = mass
*a* = acceleration in HA axis (see Figure 13)
Σ*Δs* = total change in displacement (sum of upward displacements; ‘troughs’ to ‘peaks’ on filtered acceleration plot).

**Figure 14.**
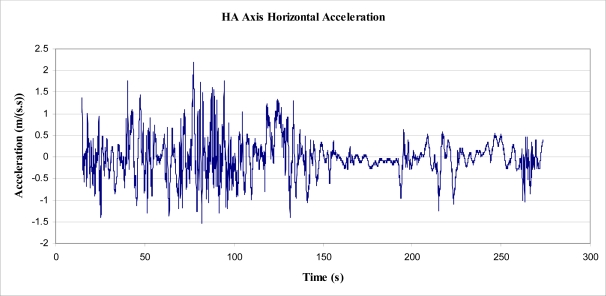
Acceleration in horizontal axis, HA (time 15 to 273 s).

It was found, however, that the abovementioned process would produce results for velocities and displacement that were highly dependent upon the cut off frequency of the high pass filter. That is, the high pass filter was used to eliminate the slow build up (drift) of velocity, as integrated from acceleration. The elimination of this build up (or drift) was directly proportional to the cut-off frequency, as was the resulting integrated displacement. It was found that the cut off frequency could be altered to give ‘realistic’ peak displacements for the tree sway, from which calculation of energy could be made. Analysis was performed on the data recorded between times 15 and 273 s (Figure 14), on the HA axis (for simplicity), which was considered representative of all of the data for that axis (Figure 7).

**Figure 15.**
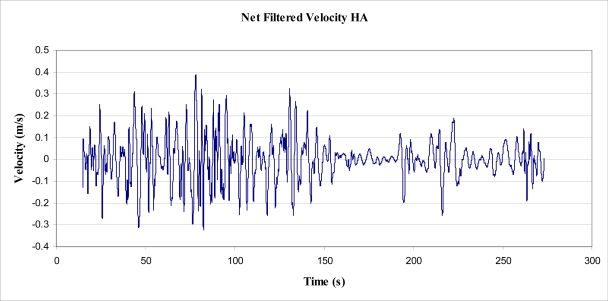
Derived, high-pass filtered velocity in horizontal axis HA (cut off 0.3 Hz).

**Figure 16.**
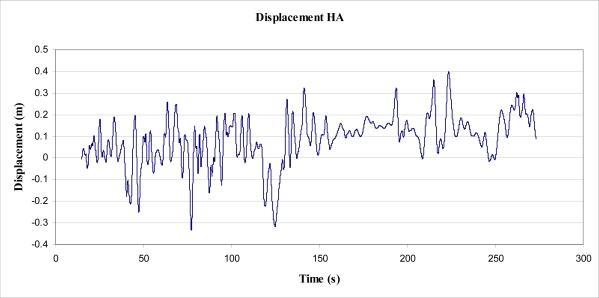
Derived displacement in horizontal axis, HA.

**Figure 17.**
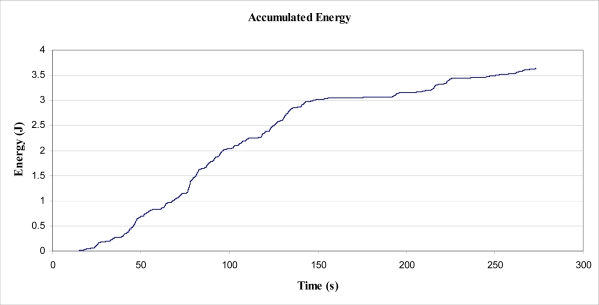
Accumulated Energy (E_MOV_) from acceleration in axis HA, for inertial mass = 5 kg.

As stated, a Fast Fourier Transformation (FFT) was performed on the velocity data, and a number of cut-off frequencies were experimented with, to view the effect on velocity and subsequent displacement (Figure 15). Cut-off frequencies of less than about 0.3 Hz produced particularly large magnitudes of displacement, such that they were unrealistic. Even the values for displacement obtained from a 0.3 Hz cut-off are larger than observed and 5 times greater than those magnitudes measured in the suspended mass experiment (Figure 16). However, it was observed (Figure 17) that the accumulated energy for this cut-off frequency was 3.7 J over a period of 258.58 s, equating to an average power output of 14 mW. This figure is, for reasons mentioned, quite optimistic. Also of note, the accumulated energy calculated was based on an inertial mass of 5 kg. The effect of an installed (inertial) mass upon the acceleration rates of the tree have not been taken into account in this determination, and no experimentation into this effect has been undertaken.

## 5. Discussion

To put the results shown above into context, examples of the energy consumption of a typical wireless sensor node are provided. The energy required to power a wireless sensor node is dependent upon a number of factors. These factors include such aspects as the electrical requirements of the various brands and types of nodes, any associated sensor power requirements, data sampling rate, radio communications quality and reliability, battery voltage, as well as any loss due to battery self-discharge. Table 2 shows the average power consumption in columns 1 to 3, and subsequent daily energy requirements in columns 4 to 6, of a Fleck3B wireless sensor node, programmed for 3 different regimes.

From the three methods of movement energy harvesting considered, it is clear that one of those methods stands out as clearly superior in terms accessing the energy available, *i.e.*, the tethered harvesting option. Using this method there is certainly enough power from the experimental tree, if harvested efficiently, to operate a Fleck3B wireless sensor node, and possibly some sensors, from a windy day. Further analysis is required to determine the energy output over a significant period of time, however, it is noteworthy that the average available power output of 44 mW was for one axis of movement and that this value would surely be larger if more directions of movement were investigated and harvested. This figure is certainly greater than the magnitudes found for either of the two methods by which an inertial harvester might generate energy, at 38 μW and the optimistic 14 mW. These values are obviously (and expectedly) far below the modeled and calculated value of 496 Watts, for a Beaufort 4 continuous wind (of 6.7 m/s). This is due to a number of reasons: the tree dissipates wind power from all parts of the tree, whereas the experiments outlined considered just one point on the trunk. Harvestable energy is a fraction of the elastic energy within the tree, and losses due to internal damping and wind vortices cannot be harvested using the methods outlines in this article.

However, the calculated Beaufort 4 wind gust tree sway energy of 9.2 J compares well to the 40.2 J of energy collected in the 900 s of sampling, where wind speeds were estimated to be as high as Beaufort 4. Further, from Figure 5, it can be seen that from time 455 s to 470 s, the tree sway reversed due to a change in wind direction or where wind died away and then returned, and that the total displacement was about 115 mm. This one sway performed work of 5.6 J to lift the mass, and is certainly of the same order of magnitude as the 9.2 J calculated using the model. As such, it believed that the model is a useful starting point for calculation of energy available within one sway of a tree, and useful to determine how much energy might be available for a tethered harvester to draw energy from.

It was difficult to correlate the wind dissipation by the tree to power available for harvesting, as it appeared to the observers of the experiment that the vast majority of the work performed by the tree on lifting the mass, was *due to changes in wind speed* (a wind gust), not the constant flow of wind as initially hypothesised. As such it was believed that further work on correlation of wind gustiness to energy/power available for energy harvesting would be more useful than attempts to correlate wind speed to power available for harvesting.

As discussed in Section 4.4, above, the energy available to an inertial energy harvester was not able to be accurately found from these experiments due to the drift in the displacement data determined from double integration of acceleration data. This resulted in no firm values for step displacement, from which energy calculations would have been derived. Data logging of trunk acceleration and displacement at the same time, would allow determination of energy available for an inertial mass harvester simpler. However, the simple analysis in this document reveals that despite some estimation problems, the energy captured from an inertial system is much less than for a tethered system. Additionally, the reported experimentation did not take into account any relationship between known wind speed and power output, simply an estimate for the wind speed (using the Beaufort scale). To more accurately estimate the energy available for a movement energy harvester, wind data will be required, for correlation.

The effect on energy output due to the size of a tree was not specifically investigated in the above work; however, verification of some of the modelling techniques used may be required. This would allow known tree properties for any tree to be used to determine an estimate of average power available.

Lastly, the point on the tree trunk used for the experiments outlined was determined somewhat arbitrarily, *i.e.*, it was approximately in the middle of the foliage, and aligned such that a string could be connected horizontally to the trunk. It was not determined where the optimum point exists on a tree, from which to connect a harvester, to maximise available energy/power. Further work could be conducted into this area to determine if harvester location is a significant factor with the aim of maximisation of energy extraction.

## 6. Conclusions

The use of sensor nodes in forests is expanding for a variety of research end uses. In most outdoor areas solar panels offer a good energy source for a sensor node. However, as discussed in this paper, the under canopy area of a forest has a much lower solar resource and this has lead to research into a variety of non-solar energy sources. The process discussed in this report is energy from tree movement.

While wind power is a good resource its direct access under trees is also limited, however, indirect access via tree movement can be exploited. This resource has been studied in terms of a number of possible methods by which energy may be extracted from tree movement, and the investigation into the amount of energy available for energy harvesting from tree movement.

The extraction methods include energy harvesting via the horizontal acceleration of the tree, the lean angle of the tree and the force/displacement of the tree, and was demonstrated by having the tree lift a mass. Device design methodologies to extract this energy was explained and it was found that energy harvesting using a force/displacement methodology showed a greater potential than the other proposed methods, as outlined in Table 3.

This paper also presented a simple model of a tree that was able to determine the power dissipation by any tree, and energy available at a given point on the tree trunk.

## Figures and Tables

**Table 1.** Data for experimental tree.

**Measured/Analysed**	**Value**	**Found from literature**	**Value**	**Calculated**	**Value**
D_MIN_	0.055 m	G	1.1	E	14.8 GPa
D_MAX_	0.111 m	Cd	0.43	D	0.056 m
L	4.16 m			k	289 N/m
A	6.4 m^2^			I	482 × 10^−9^ m^4^
P (to find k)	61 N				
y (to find k)	0.21 m				

**Table 2.** Fleck 3B energy and power consumption at 3.3 volts. Energy requirements for different regimes given; sleeping except to sample and transmit once every 1 min, sleeping except to sample and transmit once every 60 min, and as a permanent receiver.

**Operational modes (mA/mW)**			**Daily energy requirements (J)**		
Sleep	Transmit	Receive	1 min Tx	60 min Tx	Constant Rx
0.142/0.470	22/73	12/40	50	41	3,456

**Table 3.** Summary table of results, from analysis of tree movement energy harvesting methods.

	
	**Average Power**
Pendulum style inertial harvesting via tree lean	38 μW
Pendulum style inertial harvesting via tree acceleration (upper bound)	14 mW
Tethered harvesting via tree pull force/displacement	44.7 mW
Total wind power dissipated by tree	496 W
